# Scalable synthesis of high-purity TiO_2_ whiskers *via* ion exchange method enables versatile applications†

**DOI:** 10.1039/c9ra03870a

**Published:** 2019-07-30

**Authors:** Mingxu Wang, Qiang Gao, Hao Duan, Mingqiao Ge

**Affiliations:** School of Chemistry and Chemical Engineering, Yangzhou University Yangzhou 225002 PR China qianggao83@gmail.com; Key Laboratory of Eco-Textiles, Ministry of Education, Jiangnan University Wuxi 214122 Jiangsu China

## Abstract

In this work, high-purity titanium dioxide (TiO_2_) whiskers with different crystal forms were synthesized *via* ion exchange and controlled calcination methods. TiO_2_ whiskers are of 5–10 μm in length with a length-to-diameter ratio of 10–20. A systematic investigation was established to explore the hydration process of K^+^/H^+^ exchange, anatase-rutile transformation in the calcination process and the applications of TiO_2_ whiskers. Compared with the other strategies previously used for the synthesis of TiO_2_ whiskers, it was found that large-scale production was obtained under mild reaction conditions, which represented a facile and mild route for industrial production and expanded the versatile applications of TiO_2_ whiskers. Moreover, with the addition of nano TiO_2_ colloid as a special accelerant, the calcination process yielding uniform morphology and controllable crystal form was explored. Confirmatory experiments indicated that anatase and rutile TiO_2_ whiskers respectively show excellent photocatalytic activities and unique carrier performance for fabricating functional whiskers.

## Abstract 2

In this work, high-purity titanium dioxide (TiO_2_) whiskers with different crystal forms were synthesized *via* ion exchange and controlled calcination methods.

## Graphical abstract

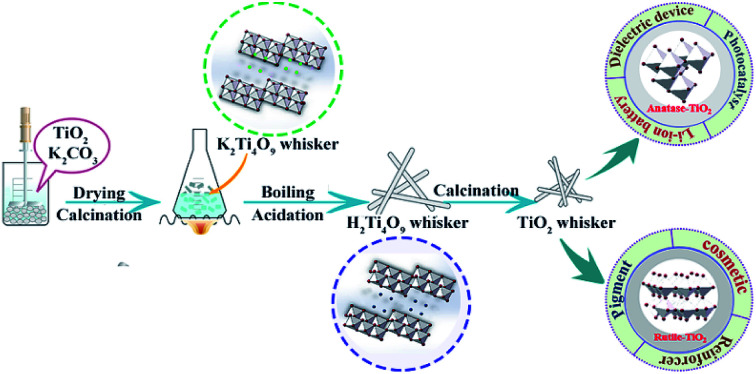

## Introduction

A whisker is a kind of single crystal with fibre-like shape. Over the past few decades, titanium dioxide (TiO_2_) whiskers have attracted the attention of many scholars for the highly ordered arrangement of atoms, which gives them excellent physical, chemical and mechanical properties.^1–3^ Anatase, rutile, plate titanium and TiO_2_(B) are the main crystal structures of TiO_2_, among which only two crystal types (anatase and rutile) have application value. TiO_2_ of anatase type is widely used in photocatalysis and semiconductor fields due to its excellent optical properties,^4–6^ while rutile TiO_2_ performs well in strengthening and toughening materials owing to its stable crystal structure and physical and chemical properties.^7,8^

Typically, TiO_2_ whiskers are prepared by various methods such as the hydrothermal method, microemulsion synthesis method, sol–gel method, vapor deposition method and ion exchange method.^9–12^ Among these methods, the ion exchange method^13^ is used to obtain pure hydrated titanic acid by the hydration reaction of the H^+^/K^+^ ion exchange with layered potassium tetratitanate whiskers as raw materials, and then TiO_2_ whiskers can be obtained *via* the calcination of hydrated titanium acid (TiO_2_·*x*H_2_O). Through this approach, large-scale production of whiskers was achieved under mild reaction conditions, overcoming the defects of high temperature, high pressure and low yield of solid-phase synthesis. However, the H^+^/K^+^ ion exchange of K_2_Ti_4_O_9_ is a multistage equilibrium process of complex reactions, and the intermediates formed under different hydration conditions are also diverse. According to the research findings of the potassium ion titration experiment reported by Sasaki,^14^ the products generated during the hydration process of ion exchange include H_2_Ti_4_O_9_·1.2H_2_O, K_0.5_H_1.5_Ti_4_O_9_·0.6H_2_O, KHTi_4_O_9_·0.5H_2_0, K_1.4_H_0.6_Ti_4_O_9_·1.2H_2_O and K_2_Ti_4_O_9_·2.2H_2_O. Therefore, in the ion exchange method, it is difficult to completely remove K^+^ ions and impossible to obtain highly purified TiO_2_ whiskers with uniform morphology by additional calcination steps. Previous studies reported that hydrochloric acid at a molar ratio of *n*(HCl)/*n*(K_2_Ti_4_O_9_) = 2 was used to substitute the K^+^ ions in the whiskers with slightly excessive H^+^ ions. When the concentration of hydrogen ions in the solution is higher than 0.05 mol L^−1^, hydrogen ions will cause Ti^4+^ to precipitate out,^15^ and thus, the layered structure of K_2_Ti_4_O_9_ will be destroyed. Meanwhile, the remaining process of ion exchange is restricted by the concentration of ions. Therefore, repeated ion exchange operations are necessary to remove the excess K^+^ ions, which results in the wastage of reagents and causes environmental pollution. Furthermore, the calcination process, which is crucial for the quality as well, including the morphology and crystal form, has not been investigated in detail in previous works.

Herein, we report a facile and effective strategy to controllably prepare high-purity TiO_2_ whiskers based on the K^+^/H^+^ exchange model built by Bao.^16^ The hydration process of ion exchange is divided into multiple stages of equilibrium. Compared with the previous studies, the method of ion exchange is implemented by controlling the pH value of the suspension, which is easy to monitor and causes little damage to the whiskers. Most importantly, the goal of full replacement of K^+^/H^+^ (>99%) is achieved. Meanwhile, in order to adapt to the requirements of different applications of TiO_2_ whiskers, different calcination temperatures of hydrated titanium acid were explored. Based on this, an accelerant (nano TiO_2_ colloid) has been used to enable the transformation of rutile TiO_2_ crystals at lower temperatures, which reduces energy consumption and avoids morphological changes to the whiskers due to the high calcination temperature. Confirmatory experiments indicated that the anatase and rutile samples respectively show excellent photocatalytic activities and stable carrier performance for fabricating functional whiskers. It can be assumed that our work is of great significance to promote the industrialization of TiO_2_ whiskers.

## Experimental

### Materials

High-purity anatase TiO_2_ nanoparticles with an average diameter of 250 ± 35 nm were purchased from Shanghai Jianghu Titanium White Product Co. Ltd. China. Anhydrous K_2_CO_3_ and HCl were obtained from Sinopharm Chemical Reagent Co. Ltd., China. Nano TiO_2_ colloid (25 nm, 20 wt%) was supplied by Fine-Blend Compatilizer Jiangsu Co. Ltd. All the chemicals and reagents used in this study were of analytical grade and were used without further purification.

### Preparation of initial materials

K_2_Ti_4_O_9_ whiskers were prepared by a controlled calcination method.^17,18^ First, anhydrous K_2_CO_3_ was mixed with anatase TiO_2_ nanoparticles at a molar ratio of *n*(TiO_2_)/*n*(K_2_CO_3_) = 3. Then 2.883 kg of K_2_CO_3_ was completely dissolved in 2.5 L deionized water. Following this, 5 kg of TiO_2_ nanoparticles and K_2_CO_3_ solution were stirred and mixed in a beaker for 3 h. The mixture was dried in an oven at 200 °C, and then ground into powder. K_2_Ti_4_O_9_ whiskers were obtained by the calcination process in a muffle furnace at 10 °C min^−1^ up to 1000 °C for 8 h. Finally, the bunchy K_2_Ti_4_O_9_ whiskers were dispersed into separated whiskers in the solution after being boiled with deionized water, and the excess K_2_O on the whisker surface was removed as well.

### K^+^/H^+^ ion exchange and preparation of TiO_2_ whiskers

**Fig. 1:**
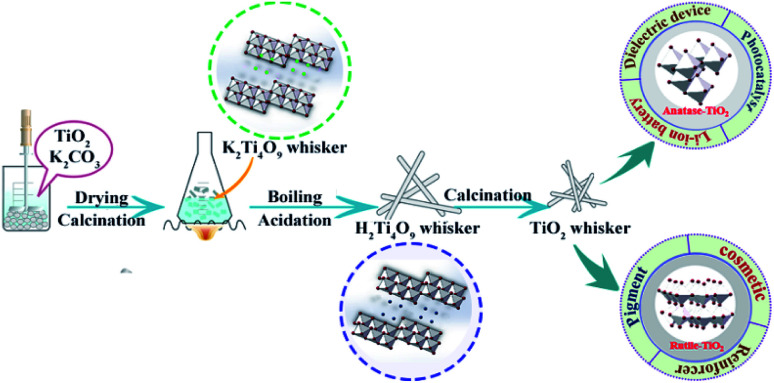
Schematic of the synthetic strategy for TiO_2_ whiskers.

Fig. 1 shows the schematic of the synthetic strategy for TiO_2_ whiskers. Dried potassium tetratitanate whisker powder (5.85 kg) was suspended in deionized water at different *V*_0_ (mass ratio, *m*H_2_O/*m*K_2_Ti_4_O_9_) degrees with the temperature remaining the same, and 1 mol L^−1^ hydrochloric acid was added into the mixture at a constant rate until its pH reached a controlled value. Meanwhile, the potassium concentration was monitored during the whole reaction process. After the K^+^/H^+^ ion exchange was over (the concentration of K^+^ or pH value of the mixture did not change within 10 min), the obtained product (hydrous titanium dioxide) was filtered and washed with deionized water to remove potassium chloride. Hydrated titanium acid can undergo a dehydration reaction at low temperatures to completely generate anatase TiO_2_; however, rutile TiO_2_ needs further heating treatment. Finally, 5 kg of TiO_2_ whiskers could be obtained. Several pilot-scale production operations indicate that our method can achieve a good utilization rate of raw materials, and the actual yield is about 92 ± 2%. The schematic flow chart and photographs of products are revealed in Fig. S1.†

To further improve the morphological quality of whiskers, nano TiO_2_ colloid was doped as an accelerant in the calcination process. Anatase TiO_2_ whiskers and nano TiO_2_ colloid were weighed with different mass ratios, mixed to form a homogeneous dispersion (taking ethyl alcohol as the solvent), and the solvents volatilized completely by using the ultrasonic bath. Finally, the sample was prepared by the calcination process under atmospheric pressure.

### Photocatalysis and whisker carrier experiments

To investigate the applicability of TiO_2_ whiskers, the experiment of photocatalysis and carrier loading was performed using TiO_2_ whiskers with different crystal structures (Fig. 1). A 110 W UV lamp was employed as the light source for the photodegradation experiments, and an aqueous solution of methylene blue dye (10 mg L^−1^) was used as the reactant. The TiO_2_ whiskers were well dispersed in the aqueous solution of methylene blue dye after stirring for 10 min, then the light was turned on and the suspension was collected (3 mL) every 15 min. The centrifuge was used to separate the supernatant from TiO_2_. Finally, the degradation percentage of the methylene blue dye was determined based on the absorbance of the obtained supernatant solution, measured using a 721 visible spectrophotometer (Shanghai Precision & Scientific Instrument Co., Ltd., China) at a wavelength of 664 nm.

A whisker carrier experiment was performed *via* a chemical coprecipitation method from our previous study.^19^ The TiO_2_ whiskers were first dispersed in deionized water and heated to 65 °C under constant stirring. Then, a quantitative SnCl_4_·5H_2_O and SbCl_3_ mixture solution was added dropwise into the suspension, and a NaOH aqueous solution was also added to keep the pH value constant at 2. Following the titration of the mixed solution, stirring and ripening for 2 h, the obtained slurry was filtered and washed to remove the residual chloride ions. Finally, after drying and calcination, TiO_2_ whiskers coated with antimony-doped SnO_2_ (ATO@TiO_2_) were prepared, and the whiteness and electroconductivity were measured.

### Characterization

In this work, ion analyzers (ST5000i/F, ST3100, OHAUS Instrument, America) were used in the measurement of the activity of K^+^ and H^+^ ions. The morphologies of whiskers were examined by scanning electron microscopy (SEM, SU-1510, Hitachi, Japan) at an acceleration voltage of 10 kV and a working distance of 10.6 mm, high-resolution transmission electron microscopy (HRTEM, Tecnai G2 F30 S-TWIN), and energy-dispersive X-ray spectrometry (EDS). The phase-transition temperature was determined by thermogravimetric analysis (TGA, TA-Q500, TA instruments, New Castle, DE, USA), which were performed on dried powders in flowing nitrogen (60 cm^3^ min^−1^) over a wide temperature range of 50–1000 °C at a heating rate of 5 °C min^−1^. The crystallographic structures of titanate and titanium dioxide were obtained using a X-ray powder diffractometer (XRD, D2 PHASER, Bruker AXS GMBH, Germany) operating in the reflection mode with Cu Kα radiation in the 2*θ* range of 5–60° at a scan rate of 0.1 s per step.

## Results and discussion

### Hydration process of K_2_Ti_4_O_9_ whiskers by K^+^/H^+^ ion exchange

During the hydration reaction of layered potassium tetratitanate whiskers, several parameters such as pH value, water quantity, temperature and hydration medium may affect the K^+^/H^+^ ion exchange.^20^ According to actual exploration and research, all the above-mentioned factors can be attributed to the pH value (ion concentration) and temperature. The pH value mainly affects the ion exchange capacity, while the temperature mainly affects the ion exchange rate. In order to avoid damages to the whisker structure caused by the high acid concentration, the pH value in the pickling process was selected from 2 to 5.

1 K_2_Ti_4_O_9_ + 2H^+^ + 1.2H_2_O → H_2_Ti_4_O_9_·1.2H_2_O + 2K^+^

**Fig. 2:**
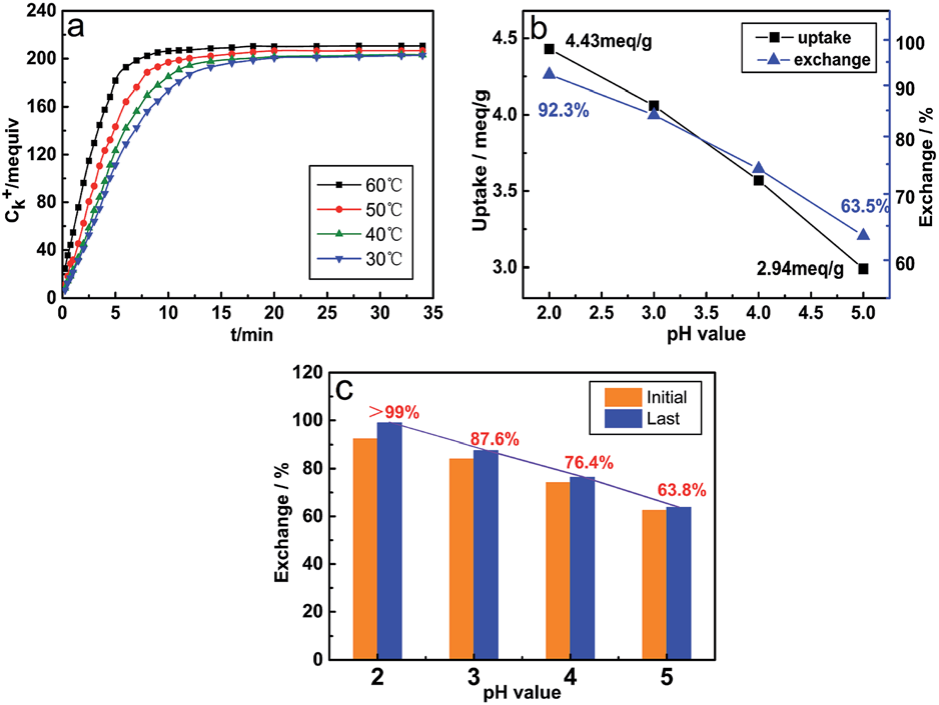
(a) Diagram of K^+^/H^+^ exchange capacity over time at pH = 2. (b) Ion exchange capacity at different pH values in the initial substitution reaction. (c) The initial and final K^+^/H^+^ exchange ratio.

**Fig. 3:**
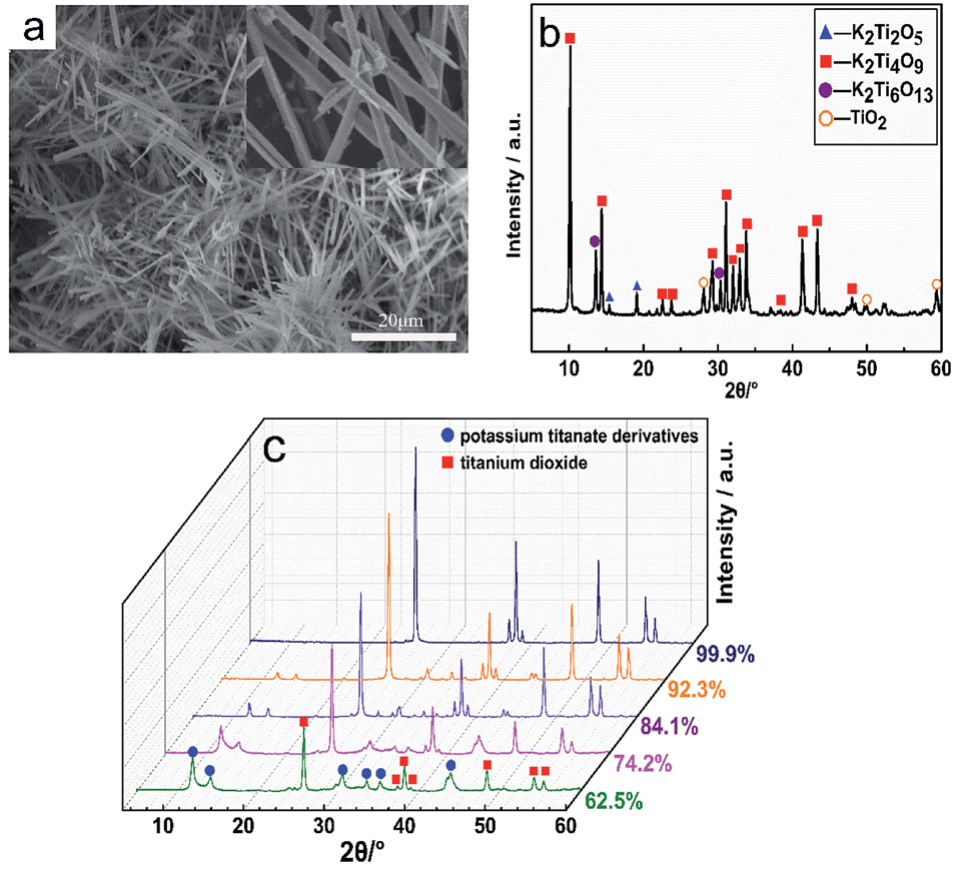
(a) SEM image of K_2_Ti_4_O_9_ whiskers. (b) XRD pattern of the initial material for ion exchange. (c) XRD patterns of the hydration process with different degrees of ion exchange after calcination at 700 °C for 2 h.

Fig. 2a reveals the variation law of the K^+^ concentration during the hydration reaction. The ion exchange reaction can be divided into three stages. In the initial stage (0–5 min), the ion exchange reaction is accelerated forward due to a large difference in the concentration of K^+^ and H^+^ ions. At this stage, the exchange capacity of ions has reached more than 80% of the total capacity. When the exchange reaction enters the second stage (7–10 min), as the ion concentration difference decreases, the rising rate of K^+^ ion concentration begins to decrease with the extension of the reaction time, and the exchange quantity slowly increases until reaching a stable value. Finally, the K^+^ ion concentration tends to level off, suggesting that the ion exchange reaction has reached the final equilibrium stage.^21^ The rising temperature has a significant positive effect on the ion exchange reaction, and it can be found that with the increase in temperature, the time to reach the equilibrium of the ion exchange reaction becomes shorter, but the influence of the temperature on the ion exchange quantity is inconspicuous. Fig. 2b shows the ion exchange capacity at different pH values in the initial substitution reaction. The pH value at equilibrium is positively correlated with the ion exchange capacity, and the ion exchange capacity is the highest at pH = 2, which is up to 92.3%. In order to further remove K^+^ ions to obtain high-purity hydrated titanic acid (TiO_2_·*X*H_2_O) whiskers, the multi-step (four steps) ion exchange reaction was conducted with *V*_0_ = 20, *V*_0_ = 20, *V*_0_ = 10 and *V*_0_ = 10, respectively. The result of the ion exchange reaction is shown in Fig. 2c. Among these different conditions, the ion exchange process is thoroughly approached (>99%) when the pH value is controlled to 2. When the pH > 2, repeated ion exchange experiments show a limited increase in the amount of ion substitution (reaching the limited value and no longer increasing), and the calcined products are TiO_2_·*X*H_2_O mixed with potassium titanate derivatives at different K/Ti ratios (Fig. 3b). Such phenomena and results are determined by the inherent equilibrium of the ion exchange reaction, wherein the solid–liquid phase reaction, which is an important factor during the initial ion exchange reaction, growth of *V*_0_ and concentration of H^+^ ions can effectively promote the reaction equilibrium to shift toward the production of H_2_Ti_4_O_9_·1.2H_2_O (especially in the acidic region, 80% of the displacement occurs in 0–5 min). However, during the process of multi-step ion exchange reactions, the rate of ion exchange is obviously slowed down. The diffusion through the inner and outer membranes replaces the solid–liquid phase reaction to become the main step of the ion exchange reaction. At this point, lowering the pH of the solution becomes the main driver for increasing ion transfer. The results indicated that only K^+^ total replacement (>99%) could be achieved under the condition of pH ≤ 2. Meanwhile, considering the damage of the layered structure (Fig. 3a) caused by very high acidity, the optimal pH value for fabricating high-purity hydrated titanic acid is maintained at 2.

Fig. 3c shows the XRD patterns of the hydration process with different degrees of ion exchange after 2 h of calcination at 700 °C. The results revealed that the characteristic peaks of anatase TiO_2_ (2*θ* = 25.4°, 37.3°, 48.1°) appear in the product, and gradually intensify with the increase in the ion exchange capacity. Meanwhile, the peak intensities of 2*θ* at 11.7°, 14.3°, 30.5° and 43.3° corresponding to the peak of potassium titanate derivatives gradually weaken, which is due to the ion exchange process, where K^+^ is gradually replaced by H^+^. Importantly, after the multi-step ion exchange, the final product after calcination is pure TiO_2_.

### Crystalline product of calcination

**Fig. 4:**
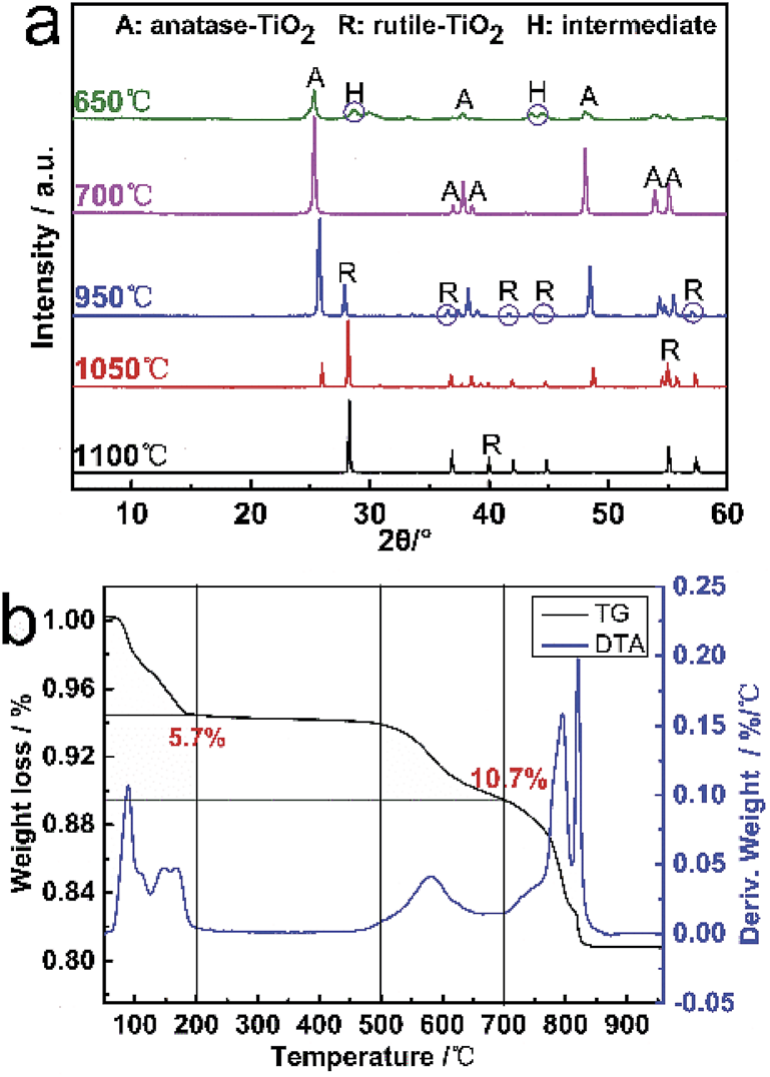
(a) XRD patterns of the products calcined by H_2_Ti_4_0_9_·1.2H_2_O at different temperatures for 2 h. (b) TG and DTA curves of H_2_Ti_4_0_9_·1.2H_2_O prepared by K^+^/H^+^ exchange.

TG and DTA curves for the hydrated titanium acid (H_2_Ti_4_0_9_·1.2H_2_O) from 50 to 1000 °C are shown in Fig. 4b. The weight loss is found to extend over a wide temperature range for the slow dehydration process, which can be mainly divided into three weight-loss steps of the sample. The first step commences around 50–200 °C, corresponding to the peak in the DTA curve, which is certainly due to dehydration. The weight loss ratio in this part is approximately 5.7%. As the temperature increases, the second step occurs at 500–700 °C, which can be related to the formation of anatase TiO_2_ from H_2_Ti_8_O_17_. At this step, the weight loss is about 4.8%. The last DTA peak at 800–1000 °C is due to the phase transformation from anatase to rutile. In terms of the entire process, the total weight-loss of H_2_Ti_4_0_9_·1.2H_2_O from 50 to 700 °C is about 10.7% (basically consistent with the theoretical calculation value of 11.01%), which contains several appearances of multiple intermediates and the transformation of various crystalline TiO_2_ (the dehydration reaction equation is shown). Consequently, the calcination temperature exerts a crucial effect on the synthesis of TiO_2_ whiskers.

2 H_2_Ti_4_O_9_·1.2H_2_O → H_2_Ti_4_O_9_ + 1.2H_2_O

3 H_2_Ti_4_O_9_ + 1.2H_2_O → 0.5H_2_Ti_8_O_17_ + 1.7H_2_O

4 0.5H_2_Ti_8_O_17_ + 1.7H_2_O → 4TiO_2_ + 2.2H_2_O

Based on the TG/DTA analysis of H_2_Ti_4_0_9_·1.2H_2_O, the TiO_2_ whiskers were prepared by calcination at high temperatures. Fig. 4a shows the XRD patterns of the products calcined by H_2_Ti_4_0_9_·1.2H_2_O at different temperatures for 2 h. With the increase in temperature, the intensity of peaks belonging to anatase TiO_2_ (25.3°, 48.0° and 53.8°) became weaker. Meanwhile, similar diffraction peaks at 2*θ* = 27.6°, 36.5°, 44.4° and 54.8° corresponding to the lattice planes of rutile TiO_2_ have appeared. Moreover, the characteristic peaks with a mountain shape of anatase TiO_2_ (36.8°, 37.8° and 38.5°) also gradually became indistinct until it disappears. Anatase TiO_2_ whiskers can be completely formed by the dehydration of H_2_Ti_4_O_9_·1.2H_2_O whiskers at 700 °C for 2 h. Meanwhile, the phase transformation of whiskers from anatase to rutile is a continuous reaction process with a temperature range of 850–1100 °C.

**Fig. 5:**
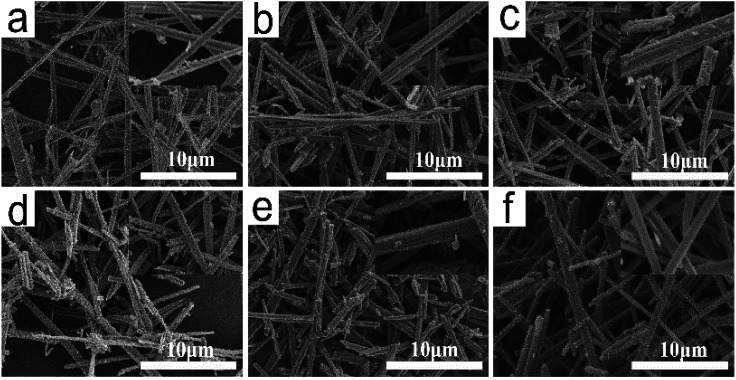
SEM images of TiO_2_ whiskers calcined at different temperatures for 2 h ((a) 600 °C, (b) 700 °C, (c) 800 °C, (d) 950 °C, (e) 1050 °C, (f) 1100 °C).

**Fig. 6:**
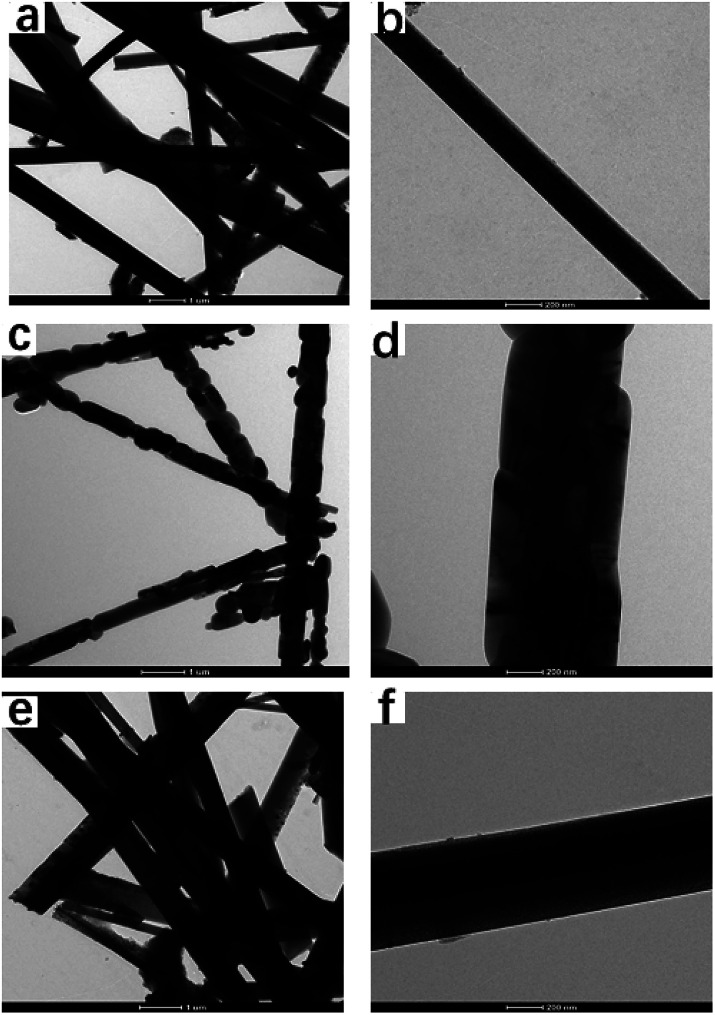
HR-TEM images of TiO_2_ whiskers calcined at different temperatures for 2 h ((a and b) 600 °C, (c and d) 850 °C, (e and f) 1100 °C).

Fig. 5 shows the SEM images of TiO_2_ whiskers after calcination at 700–1100 °C for 2 h. The processes of ion exchange and calcination both retain the layered structure and fibrous morphology of the initial whiskers. However, the change in length, diameter and surface morphology were observed after the calcination process. After the calcination at 600 °C, H_2_Ti_4_O_9_·1.2H_2_O whiskers initially form anatase TiO_2_ whiskers. With the increase in the calcination temperature and the prolongation of the holding time, pothole-shaped defects began to appear on the surface of the whiskers (Fig. 6c), and it can be clearly seen that the formation of defects accelerated in the process of calcination at 700–950 °C. However, after 950 °C, defects began to slowly disappear, and the void defects completely disappeared when the calcination temperature reached 1100 °C. The whisker surface reconstituted into the smooth state again (Fig. 5f and 6f). Moreover, the whisker length shortened from 10–30 μm of H_2_Ti_4_O_9_·1.2H_2_O whisker length to 5–15 μm of TiO_2_ whisker length. Compared with the whisker length, the change in the whisker diameter is not significant, approximately 0.1–0.2 μm.

**Fig. 7:**
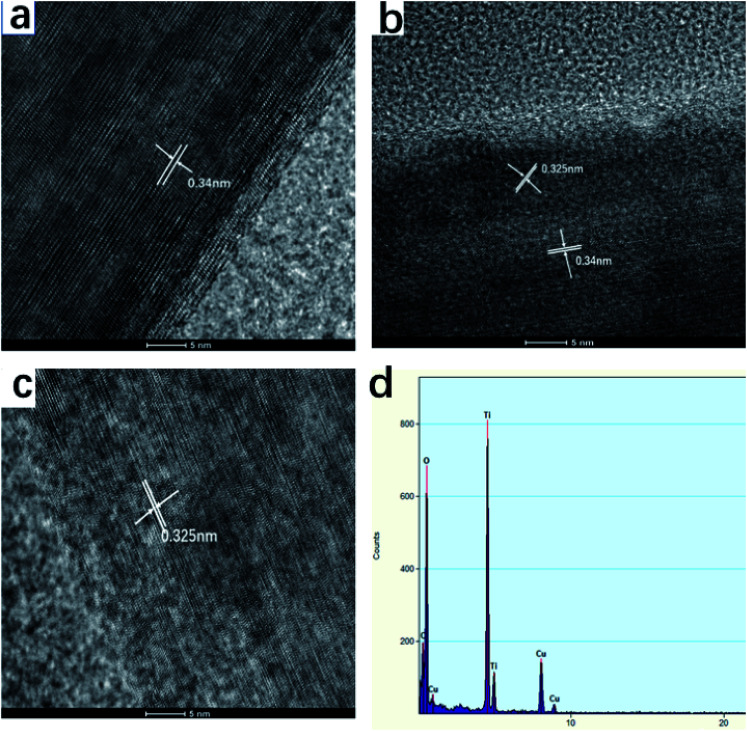
HR-TEM images and EDS spectrum of TiO_2_ whiskers calcined at different temperatures for 2 h ((a) 600 °C, (b) 850 °C, (c) 1100 °C, (d) EDS spectrum of (b)).

The TiO_2_ whiskers are randomly oriented with respect to each other according to HRTEM observation. The spacing of the fringe patterns of the TiO_2_ whiskers calcined at 600 °C (Fig. 7a) was clearly observed and was determined to be 0.34 nm, corresponding to the standard data (JCPDS 75-1537) in the (101) plane (*d* = 0.34 nm) of the TiO_2_ anatase phase. The lattice spacing is about 0.325 Å between adjacent lattice planes of the TiO_2_ whiskers calcined at 1100 °C, corresponding to the distance between (110) crystal planes of the rutile phase. Therefore, the growth directions of whiskers are concluded to be perpendicular to the (110) crystal planes. Furthermore, after calcination at 850 °C, a porous structure was observed; we can see the nanorod to be composed of large numbers of nanoparticles with indistinct polygonal shape and about 100 nm in diameter, indicating that the nanorod is polycrystalline and consists of non-directional nanoparticles.

In combination with the XRD, TEM and TG test analysis discussed above, the interpretation of the morphological changes is as follows: H_2_Ti_4_0_9_·1.2H_2_O whiskers obtained by K^+^/H^+^ substitution is a porous polymer consisting of small particles (primary single crystal unit), which forms into TiO_2_ whiskers with an incomplete structure through the dehydration reaction (500–600 °C), and then, further high-temperature calcination gradually improves the crystal shape of TiO_2_ (700–800 °C), and even results in the phase transformation of TiO_2_ crystals (850–1100 °C).^22^ All of these are the process of rearrangement and fusion of primary monocrystalline units,^23^ which eliminates small intercrystalline cracks and widens large intercrystalline gaps, thus forming potholes on the whisker surface. However, the crystalline form ultimately becomes flawless with further increase in the temperature and continuous calcining treatment, which is manifested as the disappearance of defects in the morphology. According to the above-mentioned results, uncontrollable changes in the morphology of the whiskers happen in the calcination process at high temperatures, which has a negative impact on the whisker quality. Nano TiO_2_ colloid was added as an accelerant during the calcination of H_2_Ti_4_0_9_·1.2H_2_O whiskers to promote the crystal transformation.^24^

**Fig. 8:**
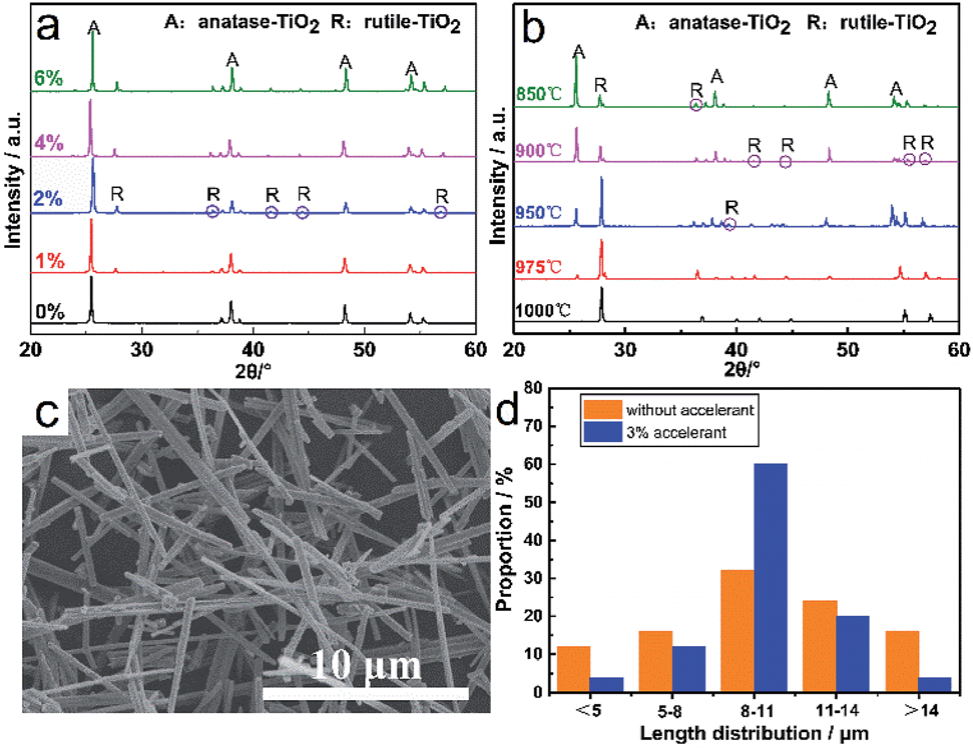
(a) XRD patterns of TiO_2_ whiskers calcined at 800 °C with different dosages of accelerants. (b) XRD patterns of TiO_2_ whiskers with 3 wt% accelerant calcined at different temperatures for 2 h. (c) SEM image of TiO_2_ whiskers with 3 wt% doped accelerant calcined at 1000 °C for 2 h. (d) Length distribution of TiO_2_ whiskers calcined at 1000 °C for 2 h before and after adding 3 wt% accelerant.

Fig. 8a shows the XRD patterns of TiO_2_ whiskers with different dosages of accelerant calcined at 800 °C for 2 h. Obviously it can be seen that there is no peak corresponding to the rutile phase in the sample without any accelerant, whereas peaks corresponding to the rutile phase appear in the sample of TiO_2_ doped with an accelerant. Additionally, the characteristic peaks gradually intensify with the increase in the accelerant content, and this promotion no longer increases significantly when the content is above 2 wt%. Fig. 8b shows the XRD patterns of TiO_2_ whiskers with 3 wt% doped accelerant calcined at different temperatures for 2 h. The results reveal that the TiO_2_ of anatase phase realizes the transformation to the rutile phase in the range of 975–1000 °C, which is lower than the transition temperature investigated previously. Fig. 8c shows the SEM images of TiO_2_ whiskers with 3 wt% accelerant calcined at 1000 °C for 2 h. Compared with the surface of pure TiO_2_ whiskers (Fig. 5d and e), it is obviously found that the surface is smooth and complete without structural defects. Moreover, the resultant TiO_2_ whiskers also exhibit good uniformity in length (Fig. 8d). The principle of induced crystallization can be attributed to the addition of seeds in the system to shorten or even cancel the nucleation process to speed up the formation of crystals. Nano TiO_2_ colloid used in this work as the accelerant is a fine rutile TiO_2_ granule, which provides a regular crystal centre and prompts crystals to grow directionally on its surface, which fundamentally explains why it improves the uniformity of the whisker morphology. Furthermore, the utilization of a seed overcomes the energy barrier that must be overcome when the nucleus is formed, which reduces the reaction activation energy, and therefore the transformation temperature is reduced, which is favorable for the mass production.

### Application of TiO_2_ whiskers with different crystal forms

**Fig. 9:**
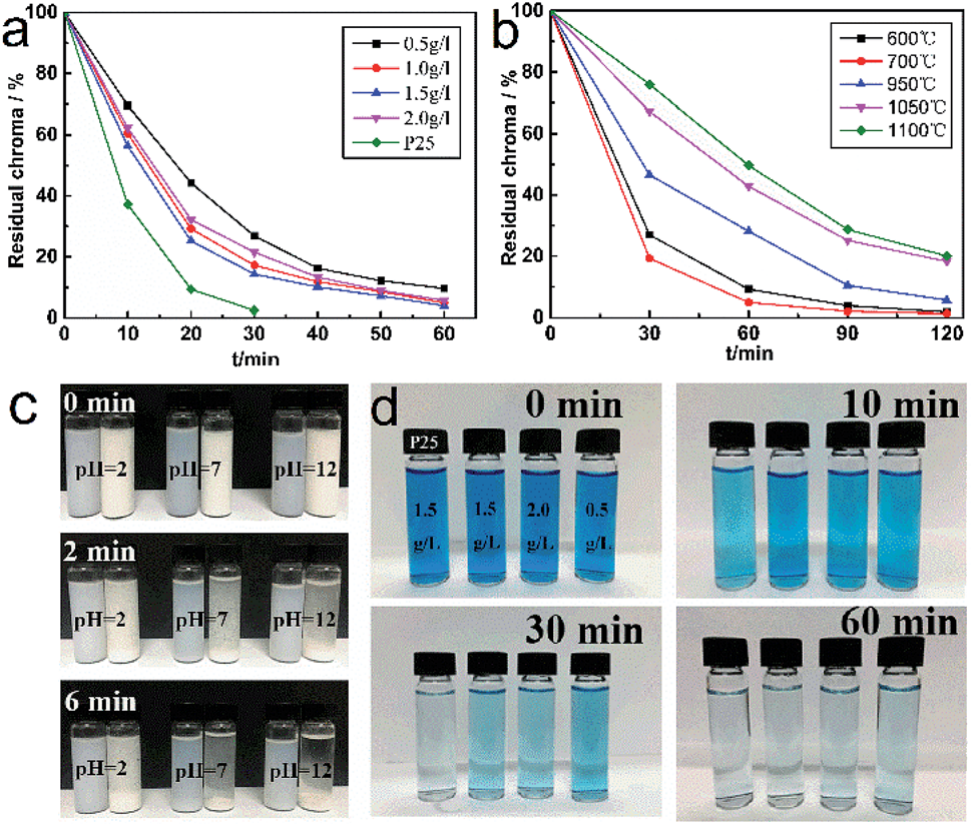
(a) Photocatalytic activity of TiO_2_ whiskers calcined at 700 °C with different addition amounts. (b) Photocatalytic activity of TiO_2_ whiskers calcined at different temperatures. (c) Settlement properties of whiskers in aqueous solutions at different pH values. (d) Digital photographs of the photocatalytic decolorization of whiskers for MB with different addition amounts.

Methylene blue (MB) was employed for representing typical organic pollutants in the adsorption test. Fig. 9 shows the photocatalytic activity of TiO_2_ whiskers with different crystal forms. Obviously, TiO_2_ whiskers obtained at 700 °C exhibit better photocatalytic performance than other samples (Fig. 9b). Compared with pure rutile TiO_2_ whiskers (79.8%), the decolorization results show that the degradation rate could reach 98.3% after 120 min. This could be attributed to the fact that the crystalline structure of rutile is more stable when it absorbs dyestuff molecules by photocatalysis, and the energy barrier required for lattice distortion on the surface is higher, which is not conducive to the reaction.^25^ Meanwhile, the difference in the energy band (CB and *E*_g_) makes the oxidation of holes in anatase TiO_2_ stronger than that in rutile.^26^

Further photocatalytic degradation experiments were conducted on MB solution with different amounts of TiO_2_ whiskers added, and it can be seen in Fig. 9a and d that the decolorization efficiency and capacity are optimized with the increase in the additive amount. When the additive amount is 1.5 g L^−1^, it performs better, and the optimization effect is not significant with further increase in the amount of addition. In addition, degussa P-25 (P25) was used for comparing the photocatalytic activity with the fabricated TiO_2_ whiskers. The results show that the degradation rate of P25 is higher than that of TiO_2_ whiskers, which is mainly attributed to its higher specific surface area and pore volume. However, it was found in the experiment that compared with P25, the micron size of TiO_2_ whiskers makes it possible to achieve better settlement performance by adjusting the pH (Fig. 9c), which facilitates the separation, purification and reuse of the catalyst from the solution.

**Fig. 10:**
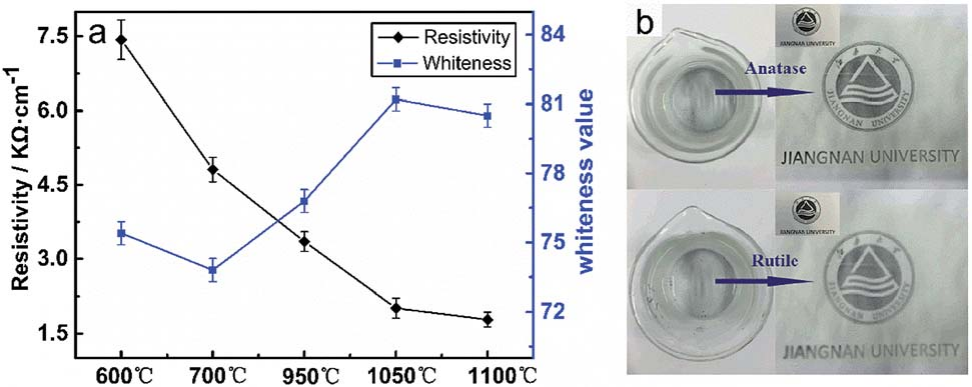
(a) Resistivity and whiteness of ATO@TiO_2_ whiskers using different TiO_2_ whiskers as the carrier. (b) Paintcoat photo of PVA with ATO@TiO_2_ whiskers as the filler.

Fig. 10a shows the results of preparing light-coloured ATO@TiO_2_ conductive whiskers with TiO_2_ whiskers as the carrier in terms of resistivity and whiteness value.^18^ The conductive whiskers using rutile TiO_2_ show a whiteness value of 80.5 ± 1.2 owing to the prominent light blocking performance of rutile TiO_2_ whiskers, which gives it a brilliant prospect in the field of functional coating. In addition, it also shows better electrical conductivity (1.78 ± 0.11 kΩ cm^−1^) than conductive whiskers with anatase TiO_2_ as the carrier. Fig. 10b is a paintcoat photo of polyvinyl alcohol with ATO@TiO_2_ whiskers as the filler (polyvinyl alcohol aqueous solution, 10 wt% dosage of ATO@TiO_2_ whiskers in polyvinyl alcohol, film thickness of 50 μm). It can be found that the covering performance and whiteness value of the paintcoat by using rutile TiO_2_ whiskers coated by ATO layer are better, which is consistent with the whiteness of ATO@TiO_2_ whiskers by using different TiO_2_ whiskers as the carrier. All these can be speculated to be due to the following reasons: during the process of loading a conductive layer by the chemical coprecipitation method, the strong acid dispersion treatment of the carrier has a negative effect on the surface of TiO_2_ whiskers, which reduces the carrying capacity of the conductive material. Besides, compared with the anatase TiO_2_ whiskers, whiskers of rutile type show better reuse value owing to its excellent chemical stability.^27^

## Conclusions

In summary, based on the K^+^/H^+^ exchange model, a facile fabrication method to prepare high-purity TiO_2_ whiskers has been described, in which hydrated titanate whiskers are subjected to acidic treatment for 4 times at a pH value maintained at 2 and a *V*_0_ value adjusted to 20, 20, 10 and 10. Moreover, by controlling the calcination temperature, the transformation reaction of different crystal forms of TiO_2_ whiskers can be achieved. Especially, with the combination of nano TiO_2_ colloid as the accelerant, the temperature range of this transformation can be reduced. The morphology and uniformity of whiskers also improved. Additional experiments were performed to investigate the applications of TiO_2_ whiskers with different crystal forms. The results show that anatase TiO_2_ whiskers exhibit a good photocatalytic activity, which can be used as the decolorization catalyst for wastewater treatment, while whiskers with rutile phase demonstrate a brilliant prospect in the field of functional coatings as a carrier owing to its optical performance and chemical stability.

## Conflicts of interest

There are no conflicts to declare.

## Supplementary Material

RA-009-C9RA03870A-s001
