# Supplementary material for: Scalable synthesis of high-purity TiO2 whiskers via ion exchange method enables versatile applications
Source: RSC Adv. 2019 Jul 30;9(41):23735–43. doi: 10.1039/c9ra03870a (PMC9069495; doi:10.1039/c9ra03870a)
Supplement: RA-009-C9RA03870A-s001 [file RA-009-C9RA03870A-s001.pdf]

Electronic Supplementary Material (ESI) for RSC Advances.
This journal is © The Royal Society of Chemistry 2019

Supporting Information

Scalable synthesis of high-purity TiO_2 whiskers via ion exchange method enables versatile applications

Mingxu Wang, [a, b] *Qiang Gao,* *[a, b] *Hao Duan*[a] *and Mingqiao Ge*[b]

[a]*School of Chemistry and Chemical Engineering, Yangzhou University, Yangzhou 225002, China*
[b]*Key Laboratory of Eco-Textiles, Ministry of Education, Jiangnan University, Wuxi 214122, Jiangsu, China.*

Corresponding author: *Dr. Qiang Gao(qianggao83@gmail.com)*

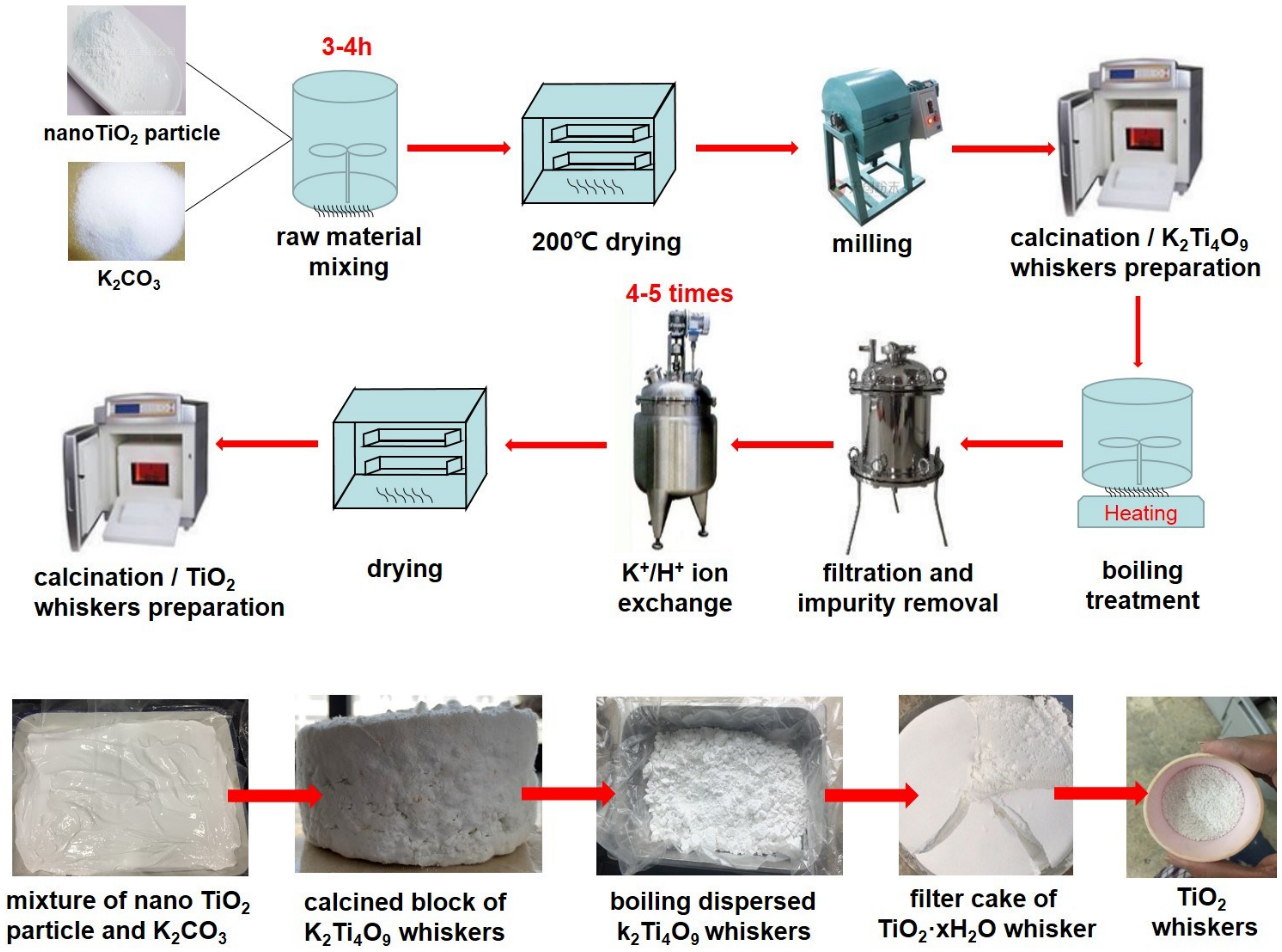

Figure S1. Schematic flow chart for the preparation of 5 kg of TiO_2 whiskers and photographs of products during the process.
